# Successes and Challenges for Diagnosis and Therapy of Acute Leukemia

**DOI:** 10.1155/2019/3408318

**Published:** 2019-11-22

**Authors:** Annalisa Lonetti, Ilaria Iacobucci, Riccardo Masetti

**Affiliations:** ^1^“Giorgio Prodi” Interdepartmental Cancer Research Centre, University of Bologna, Bologna, Italy; ^2^Department of Pathology, St. Jude Children's Research Hospital, Memphis, TN, USA; ^3^Pediatric Hematology-Oncology Unit, Department of Medical and Surgical Sciences DIMEC, University of Bologna, Bologna, Italy

Acute leukemia (AL) is a family of blood cancers that arises from the malignant transformation of hematopoietic cells of lymphoid (acute lymphoblastic leukemia (ALL)) or myeloid (acute myeloid leukemia (AML)) origins. While ALL occurs mostly in children, AML is mostly a disease of older adults, although it also peaks in infants (children aged 0-1 years). Treatment of AL remains challenging across age, with standard therapeutic treatments allowing 5-year overall survival rates of about 70% and 90% in children and 30–40% in adults, for AML and ALL, respectively. The major cause of treatment failure is relapse, and the prognosis for relapsed AL is extremely poor. In recent years, many efforts to improve the prognosis for these patients resulted in profound changes in supportive care and minimal residual disease monitoring. In addition, significant achievements have been made in our understanding of the genetic basis of both lymphoid and myeloid leukemias, which in turn allowed a better risk-adapted patients' stratification and the identification of novel biomarkers suitable for innovative targeted therapy-based approaches. We are pleased to introduce this special issue that contains original research as well as review articles covering several aspects of AL biology and clinical management.

Two interesting manuscripts in this special issue focus on the complexity of AML biology, which has implications for therapeutic intervention. L. Handschuh provided a comprehensive review of the molecular alterations of AML and their use for AML subtype classification, risk stratification, outcome prediction, and novel therapeutic approaches. In addition, novel findings identified by applying next-generation sequencing approaches were detailed. They include novel chimeric or alternative spliced transcripts, as well as small RNAs, piwi-interacting RNAs, small nucleolar RNAs, long noncoding RNAs, and circular RNAs. All these functional transcripts dramatically impair expression and increase the genetic heterogeneity of AML. The second review by P. Bernasconi and O. Borsani focused on AML stem cell-niche and the possible interventions that might affect the cross talk between leukemic cells and bone marrow microenvironment, aimed to restore the normal niche ecology and to sensitize AML cells to chemotherapy, thus overcoming niche-mediated drug resistance.

Three further manuscripts investigating genetic alterations or innovative therapeutic approaches in AML are included in this special issue. In the research article by N. Niktoreh et al., the prognostic relevance of co-occurrence of *WT1* mutations, *FLT3-ITD,* and *NUP98-NSD1*, was re-evaluated in a contemporary pediatric AML trial. Analysis of a large cohort of pediatric AML patients treated in Germany with AML-BFM protocols between 2004 and 2017 confirmed that co-occurrence of these three alterations defines a subgroup of AML patients with dismal outcome. The original research article by S. Bruno et al. reported the identification of two novel alterations in *DNMT3A* gene in two adult AML patients, a single nucleotide variant (p.Trp795Ter) and a 35 nucleotides insertion (p. Thr862_Glu863fsins), both resulting in a premature STOP codon. The authors demonstrated that both these mutations affect DNMT3A protein expression and DNMT3A-mediated DNA methylation. Finally, the research article by A. Vitkevičienė et al. suggested the addition of 3-deazaneplanocin A (HMT inhibitor) and belinostat (HDAC inhibitor) to the conventional therapy (idarubicin and retinoic acid) of acute promyelocytic leukemia (APL) patients, based on enhanced efficacy observed in their *in vitro* study.

The manuscript of C. Grobbelaar and A. M. Ford described the roles of different miRNAs in pediatric ALL, particularly focusing on their role for ALL classification, risk stratification, prediction of therapy response, and potential therapeutic targets. The authors also reported an interesting section about the recently identified gut miRNAs that likely participate in shaping the gut microbiota and might be useful as potential noninvasive diagnostic and prognostic biomarkers, or even as tools for prevention of certain subtypes of childhood ALL.

Involvement of central nervous system (CNS) by leukemia is a relatively common event and it is still a major clinical issue. To target malignant cells in the CNS, intrathecal (IT) chemotherapy is commonly used. Intrathecal therapy associates with acute and long-term neurotoxicity, as it is well documented in pediatric patients but data about long-term toxicity of IT in adults are lacking. D. M. Byrnes et al. investigated the effects of IT in adult patients, including ALL patients, who were treated over a two-year period. The results of this retrospective study demonstrated a high frequency of neurological complications secondary to IT therapy, suggesting that less toxic forms of therapy may be warranted.

Finally, E. Ledesma-Martínez et al. reviewed the biological effects of caseins, the main milk proteins, and peptides released during their digestion, focusing in particular on their interaction with the immune system, normal hematopoietic and leukemic cells. Although few researches have explored the role of caseins as antileukemic agents, what emerges is the influence of dietary intake of foods on cancer development or predisposition, sustaining the importance to pay attention on patients' nutrition during leukemia therapy.

We hope that the articles included in this special issue will provide the opportunity to deepen some aspects of acute leukemias and will stimulate future researches in the field.

